# The conserved N-terminus of human rhinovirus capsid protein VP4 contains membrane pore-forming activity and is a target for neutralizing antibodies

**DOI:** 10.1099/jgv.0.000629

**Published:** 2016-12-15

**Authors:** Anusha Panjwani, Amin S. Asfor, Tobias J. Tuthill

**Affiliations:** ^1^​ The Pirbright Institute, Ash Road, Pirbright, Woking GU24 0NF, UK

**Keywords:** virus cell entry, non-enveloped virus, picornavirus, membrane permeability, viroporin, myristoylation

## Abstract

Human rhinovirus is the causative agent of the common cold and belongs to the non-enveloped picornavirus family. A trigger such as receptor binding or low pH initiates conformational changes in the capsid that allow the virus to attach to membranes and form a pore for the translocation of viral RNA into the cytoplasm. We previously showed that recombinant capsid protein VP4 was able to form membrane pores. In this study, we show the N-terminus but not C-terminus of VP4 formed pores with properties similar to full-length VP4 and consistent with the size required for transfer of RNA. Sera against the N-terminus but not C-terminus of VP4 were shown to neutralize virus infectivity. Together, this suggests that the N-terminus of VP4 is responsible for membrane activity. This study contributes to an improved understanding of the mechanisms for involvement of VP4 in entry and its potential as an antiviral target.

Human rhinoviruses (HRVs) are ubiquitous seasonal pathogens and are the major causative agents of the common cold but are also associated with exacerbations of asthma and chronic obstructive pulmonary disease, severe bronchiolitis in infants and fatal pneumonia in elderly and immunocompromised adults (Jacobs *et al.*, 2013). Despite decades of efforts into the development of vaccines and antivirals, no effective treatment is currently available.

The picornavirus family includes significant pathogens affecting humans and animals. HRV is classified in the *Enterovirus* genus and is closely related to poliovirus (PV) and other enteroviruses such as enterovirus 71 (EV71) which has emerged as a significant public health threat in China and South East Asia in the last decade (Sabanathan *et al.*, 2014).

Picornaviruses are non-enveloped viruses and contain a single-stranded, positive-sense RNA genome which is approximately 7500 nt in length. The enterovirus capsid has icosahedral symmetry, has a diameter of approximately 30 nm and is composed of 60 copies of each of the capsid proteins VP1, VP2, VP3 and VP4. VP1, VP2 and VP3 are the major components of the capsid, while VP4 is a small (approximately 7 kDa) internal protein which lies on the inside surface of the capsid around the fivefold axes of symmetry where it is thought to stabilize interactions between pentameric capsid subunits (Ren *et al.*, 2013; Wang *et al.*, 2012). The N-terminus of VP1 is also internal to the capsid (Ren *et al.*, 2013). However, the enterovirus capsid is dynamic and undergoes ‘breathing’ at physiological temperature which involves the transient exposure of the N-termini of VP4 and VP1 (Lin *et al.*, 2012; Li *et al.*, 1994). During entry into a host cell, the appropriate trigger such as receptor binding or low pH leads to the irreversible externalization of VP4 and the N-terminus of VP1 from the enterovirus capsid (the altered particle), which are then thought to be involved in membrane interactions and pore formation for the delivery of the genome into the cytoplasm (Davis *et al.*, 2008; Fricks & Hogle, 1990; Panjwani *et al.*, 2014; Strauss *et al.*, 2013; Tuthill *et al.*, 2006). The VP1 N-terminus can be visualized at the inside surface of the native capsid in sub-3 Å resolution crystal structures of human rhinovirus (Hadfield *et al.*, 1997) and the EV71 (Wang *et al.*, 2012). It is externalized at the capsid surface in a 3 Å resolution crystal structure of the coxsackievirus A16 altered particle (Ren *et al.*, 2013) and in a 5.5 Å resolution cryo-electron microscopy reconstruction of the PV altered particle (Butan *et al.*, 2014) where it is predicted to form an amphipathic helix (Bubeck *et al.*, 2005; Fricks & Hogle, 1990) consistent with its ability to interact with membranes. In contrast, VP4 is released from the altered particle and is, therefore, not visible in high-resolution structures of such particles, and the conformation it adopts outside of the native particle remains unknown. A schematic diagram describing the externalization of the N-termini of VP1 and VP4 from the capsid is shown in the study by Ren *et al.* (2013).

The N-termini of VP4 (VP4N) and VP1 (VP1N) are exposed during capsid breathing. A previous study showed that antibodies in sera raised against VP4N peptides were able to neutralize HRV infectivity, showing that dynamic capsid alterations could be targeted by antibodies (Katpally *et al.*, 2009). In the current study, antibodies were purified using protein G chromatography from sheep sera raised against purified HRV16 or 16-mer peptides corresponding to the N (VP4N) or C (VP4C) termini of VP4 or the N-terminus of VP1 (41-mer, VP1N). The ability of these antibodies to neutralize HRV16 was compared using a one-dimensional virus neutralization assay (Rweyemamu, 1984) which measured the minimum amount of IgG required to neutralize 100 TCID_50_ of HRV16 (where 1 TCID_50_ is the dose required to infect 50 % of replicate tissue cultures).

For the antibodies raised against HRV16 and the pre-immune (control) sera, these values were 0.7 and 675 µg, respectively, indicating that antibodies in sera raised against HRV16 had neutralizing activity as expected (Fig. 1a). For the antibodies to VP4N, VP1N and VP4C, the values were 17.3, 15.7 and 830 µg, respectively, indicating that antibodies to the N-terminus of VP4 and VP1, but not to the C-terminus of VP4, also had neutralizing activity (Fig. 1a). The neutralization of HRV16 infectivity with antibodies against VP4N and the whole virus is in agreement with previous findings (Katpally *et al.*, 2009). In addition, the neutralization of HRV16 infectivity with antibodies against VP1N is in agreement with similar studies carried out using antibodies against the N-terminus of VP1 of PV (Li *et al.*, 1994).

**Fig. 1.**
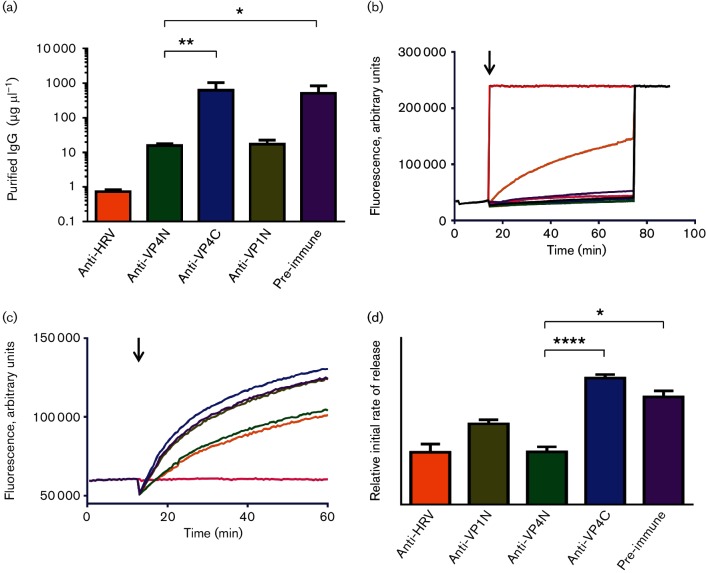
Antibodies raised against the N-terminus of VP4 neutralize HRV16 infectivity and reduce virus-induced permeability in model membranes. (a) Virus neutralization showing the minimum concentration of IgG (purified from antisera as indicated) required to neutralize 100 TCID_50_ of HRV16. Error bars represent standard error of the mean of values from four experiments. Asterisks indicate statistical significance calculated by one-way ANOVA (*P<0.05, **P<0.005). (b) Carboxyfluorescein (CF) release assays showing membrane permeability induced by 1 µg HRV16 (shown in orange) or 0.5 % Triton X-100 (shown in red). Membrane permeability induced by 1 µg pre-heated HRV16 (pink) or purified IgG from pre-immune sera (purple) or sera raised against the N-terminus of VP4 (green), C-terminus of VP4 (blue), N-terminus of VP1 (olive) or whole virus (grey) was close to baseline. At the end of the read, total release of CF was induced by the addition of 0.5 % Triton X-100 to all samples. The black arrow indicates time of addition of sample at the start of the assay. (c) CF release assays showing HRV16-induced membrane permeability after incubation of virus with purified IgG from pre-immune sera (shown in purple) or sera raised against the N-terminus of VP4 (anti-VP4N, shown in green), C-terminus of VP4 (anti-VP4C, shown in blue), N-terminus of VP1 (anti-VP1N, shown in olive green) or whole virus (anti-HRV, shown in orange). Baseline shown in pink. The black arrow indicates time of addition of sample at the start of the assay. (d) Initial rates of dye release generated using the first four data points from experiments as in (c). The colour scheme is the same as in (c). Error bars represent standard error of the mean of values from three independent experiments. Asterisks indicate statistical significance calculated by one-way ANOVA (*P<0.05, ****P<0.00005).

Capsid components VP4 and VP1N are known to be involved in membrane interactions required for virus entry. We proposed that the mechanism of neutralization by anti-VP4N and anti-VP1N antibodies involved blocking VP4 or VP1 membrane interactions. To test this, the ability of the antibodies to prevent HRV16-induced membrane permeability was investigated by incubating purified virus with antibodies at 37 °C for 1 h before mixing with small unilamellar vesicles (or liposomes) in an established carboxyfluorescein (CF) dye release assay (Davis *et al.*, 2008). The liposome composition was phosphatidic acid/phosphatidylcholine/cholesterol at 45 : 45 : 10 molar ratio. Upon addition of HRV16 to liposomes, the expected membrane permeability was induced as demonstrated by the release and unquenching of CF (Fig. 1b). HRV16 that had been pre-heated at 60 °C for 10 min (conditions known to produce empty particles which have lost VP4) were not capable of inducing dye release in this assay (Fig. 1b). Upon addition to liposomes of HRV16 pre-incubated with antibodies raised against VP4N or whole capsid, the induction of membrane permeability was reduced significantly compared with that induced by HRV16 (Fig. 1c, d). In contrast, HRV16 pre-incubated with antibodies against VP4C or VP1N induced membrane permeability comparable to that seen with HRV16 pre-incubated with pre-immune sera (Fig. 1c, d).

This confirms that the likely mechanism for virus neutralization by antibodies against VP4N involves preventing virus-induced membrane permeability. The ability of VP4N but not VP4C antibodies to reduce virus-induced membrane permeability also suggests that the N-terminus of VP4 is the component of VP4 that mediates membrane permeability. The lack of effect by anti-VP1 antibodies was unexpected given that the same antibodies had neutralizing ability and that VP1N has been shown previously to tether virus particles to liposome membranes (Tuthill *et al.*, 2006). This result could be explained by the VP4-induced membrane permeability being independent of VP1N-induced membrane interactions in this assay. For example, perhaps VP4 is released from particles to permeabilize the liposomes in the absence of the particles being tethered to the membrane by the proposed function of VP1N.

In previous studies, we reported the ability of recombinant HRV16 VP4 to interact with membranes and induce permeability by the formation of size-selective pores (Panjwani *et al.*, 2014; Davis *et al.*, 2008). In the current study, experiments to determine which part of VP4 is responsible for inducing membrane permeability were carried out using the permeability assay described above and peptides representing the N- or C-terminus of VP4. Peptides were synthesized (Peptide Protein Research) without modifications except for the addition of six lysines at the C-terminus to improve solubility and the addition of an N-terminal myristoyl group where indicated. Upon addition of 0.1 µM peptide representing the N-terminus of VP4 (VP4N-45mer – GAQVSRQNVGTHSTQNMVSNGSSLNYFNINYFKDAASSGASRLDFKKKKKK) to liposomes (lipid/peptide molar ratio of 500 : 1), extensive membrane permeability was induced as demonstrated by the release and unquenching of CF in Fig. 2(a). Addition of 0.1 µM peptide corresponding to the C-terminus of VP4 (VP4C-45mer – FNINYFKDAASSGASRLDFSQDPSKFTDPVKDVLEKGIPTLQSLEKKKKKK) did not induce permeability. This shows that the N-terminus, and not the C-terminus, is responsible for the ability of VP4 to induce membrane permeability. In order to confirm that the activity of VP4N-45mer was dependent on the amino acid sequence, a sequence-scrambled version of this peptide was also tested and did not induce permeability in the dye release assay. In addition, the role of the VP1 N-terminus in membrane permeability was also investigated using a peptide (VP1N-24mer – NPVERYVDEVLNEVLVVPNINQSHKKKKKK), but this peptide was not able to induce permeability (Fig. 2a). To understand the mechanism for permeability induced by VP4N-45mer, peptide-induced release of FITC-labelled dextrans of different sizes from within liposomes was measured using an established assay (Panjwani *et al.*, 2014). VP4N-45mer induced the release of small (4 and 10 kDa) but not large (70 and 250 kDa) dextrans (Fig. 2b), indicating that permeability by VP4N-45mer was via a discrete size-selective pore. This was in agreement with previous findings for full-length recombinant VP4 (Panjwani *et al.*, 2014) and predicts a lumen diameter of ≤12 nm, consistent with the size required for passage of single-stranded nucleic acid. Peptides VP4C-45mer, VP1N-24mer and the scrambled version of VP4N-45mer did not induce significant release of any of the FITC-labelled dextrans as shown in Fig. 2(b).

**Fig. 2.**
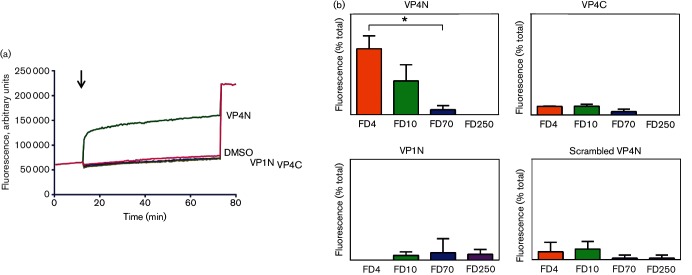
A peptide corresponding to the N-terminus of VP4 induces size-selective permeability in model membranes. (a) CF release assays showing membrane permeability induced by peptides VP4N-45mer (VP4N), VP4C-45mer (VP4C), VP1N-24mer (VP1N) or DMSO (carrier, 0.1 % final concentration). All peptides were used at 0.1 µM final concentration. Data shown are representative of three independent experiments. The black arrow indicates time of addition of sample at the start of the assay. (b) Release from liposomes of FITC-labelled dextrans of 4 kDa (FD4), 10 kDa (FD10), 70 kDa (FD70) or 250 kDa (FD250) by peptides VP4N-45mer (VP4N), VP4C-45mer (VP4C), VP1N-24mer (VP1N) or a version of VP4N-45mer with scrambled amino acid sequence (scrambled VP4N), respectively. Data are presented as percentage of total release observed by lysis of liposomes by addition of detergent. Error bars represent standard error of the mean of values from three independent experiments. Asterisk indicates statistical significance calculated by one-way ANOVA (*P<0.05).

In many picornaviruses, VP4 is co-translationally myristoylated by the covalent addition of myristic acid to the N-terminal glycine. It is important for particle assembly and stability in both native (Marc *et al.*, 1989; Moscufo *et al.*, 1991; Moscufo & Chow, 1992) and recombinant systems (Goodwin *et al.*, 2009; Abrams *et al.*, 1995). To understand the effect of myristoylation on the membrane permeability induced by VP4N-45mer, myristoylated and unmyristoylated peptides were tested in the CF liposome dye release assay described above. Permeability induced by both forms of the peptide was concentration dependent, but the myristoylated peptide induced more extensive permeability relative to the unmyristoylated peptide, as demonstrated by the release and unquenching of CF in Fig. 3(a). The size selectivity of the peptide-derived pore was not altered by myristoylation, as shown by the release of FD4 and FD10, but not FD70 and FD250 in Fig. 3(b). This finding suggests that myristoylation enhances the targeting of VP4 to membranes but does not influence the mechanism underlying formation of the membrane pore.

**Fig. 3.**
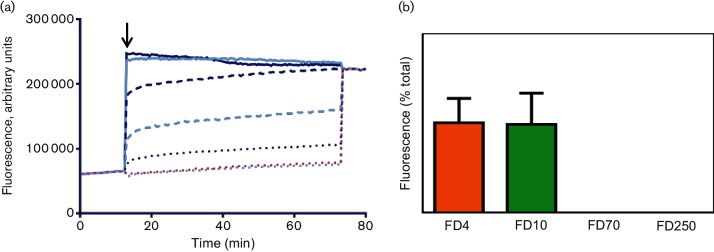
VP4N-induced membrane permeability is enhanced by myristoylation. (a) CF release assays showing membrane permeability induced by myristoylated VP4N-45mer at 1 µM (solid line, dark blue), 0.1 µM (dashed line, dark blue) or 0.01 µM (dotted line, dark blue) or unmyristoylated VP4N-45mer at 1 µM (solid line, light blue), 0.1 µM (dashed line, light blue) or 0.01 µM (dotted line, dark blue). Data shown are representative of three independent experiments. The black arrow indicates time of addition of sample at the start of the assay. (b) Release from liposomes of FITC-labelled dextrans of 4 kDa (FD4), 10 kDa (FD10), 70 kDa (FD70) or 250 kDa (FD250) by 0.1 µM myristoylated VP4N-45mer. Data are presented as percentage of total release observed by lysis of liposomes by addition of detergent. Error bars represent standard error of the mean of values from three independent experiments.

The experiments described in this study demonstrate that the ability of VP4 to permeabilize membranes resides in the N-terminus of the protein. The permeability induced by VP4N-45mer peptide in the current study was broadly comparable with that seen in previous studies with full-length recombinant VP4 (Panjwani *et al.*, 2014), both in terms of kinetics of permeability and size exclusion. The VP4N-45mer peptide may, in fact, be more active than the full-length protein due to being more soluble. Antibodies against the N-terminus but not the C-terminus of VP4 have the ability to neutralize the virus. Taken together, these findings suggest the mechanism of neutralization by these antibodies is by preventing interaction between VP4N and the membrane. Therefore, the N-terminus of VP4 may be suitable for exploration as an epitope for the induction of function-blocking antibodies to prevent or control infection.

These findings show that the N-terminal region of VP4 can make a membrane pore in the absence of other viral components. However, interactions between virus particles and the membrane are thought to involve further components such as the N-terminus of VP1 which along with VP4 are proposed to form an ‘umbilicus’ structure linking the particle to the membrane (Strauss *et al.*, 2013). This extended structure may link a membrane pore formed by the N-terminus of VP4 to the particle for delivery of the RNA through the membrane.
